# Determining independence and associations among various cardiovascular disease risk factors in 9-12 years old school-children: a cross sectional study

**DOI:** 10.1186/s12889-022-14035-6

**Published:** 2022-08-30

**Authors:** Abdulrahman I. Alaqil, Erich J. Petushek, Yuba R. Gautam, Karin A. Pfeiffer, Joseph J. Carlson

**Affiliations:** 1 Department of Physical Education, College of Education, King Faisal University, Al-Ahsa, 31982 Saudi Arabia; 2 Department of Sports Science and Clinical Biomechanics, University of Southern Denmark, Campusvej, 55, 5230 Odense, Denmark; 3 Department of Cognitive and Learning Sciences, Michigan Technological University, 1400 Townsend Drive, Houghton, MI 49931 USA; 4 Health Research Institute, Michigan Technological University, 1400 Townsend Drive, Houghton, MI 49931 USA; 5 School of Health and Human Performance, College of Health Science and Professional Studies, Northern Michigan University, 1401 Presque Isle Ave, Marquette, MI 49855 USA; 6 Department of Kinesiology, Michigan State University, 308W Circle Dr, East Lansing, MI 48824 USA; 7 Department of Radiology, Division of Sports and Cardiovascular Nutrition, Michigan State University, East Lansing, MI USA

**Keywords:** Cholesterol, Blood pressure, Waist circumference, Risk factors

## Abstract

**Background:**

Cardiovascular disease (CVD) risk assessment of children typically includes evaluating multiple CVD risk factors some of which tend to correlate each other. However, in older children and young adolescents, there are little data on the level of independence of CVD risk factors. The purpose of this study was to examine the relationships among various CVD risk factors to determine the level of independence of each risk factor in a sample of 5^th^-grade public school students.

**Method:**

A cross-sectional analysis of 1525 children (856 girls and 669 boys; age: 9-12 years) who participated in baseline CVD risk assessment for the (S)Partners for Heart Health program from 2010 - 2018. Thirteen CVD risk factor variables were used in the analysis and included blood lipids [low-density lipoprotein (LDL), high-density lipoprotein (HDL), total cholesterol (TC), and triglycerides], resting systolic and diastolic blood pressure (BP); anthropometrics [height, weight, body mass index (BMI), % body fat, waist circumference (WC)]. Additionally, acanthosis nigricans (a marker insulin resistance and diabetes), and cardiorespiratory fitness (VO2 ml/kg) was estimated using the *PACER.* Descriptive statistics, bivariate Pearson correlations, and principal component analysis were used to determine the relationships among these variables and the independence.

**Results:**

Parallel analysis indicated two components should be extracted. Among the two components extracted, WC, % body fat, and BMI loaded highest on component 1, which explained 34% of the total variance. Systolic BP and diastolic BP loaded predominantly on component 2 and accounted for 17% of the variance. Cardiorespiratory fitness, acanthosis nigricans, HDL, and triglycerides loaded highest on the first component (loadings between 0.42 and 0.57) but still suggest some non-shared variance with this component. Low-density lipoprotein had low loadings on each component. Factor loadings were stable across sex.

**Conclusion:**

Among the various CVD risk indicators, measures of adiposity loaded highest on the component that explained the largest proportion of variability in the data reinforcing the importance of assessing adiposity in CVD risk assessment. In addition, blood pressure loaded highest on the second component, suggesting their relative independence when assessing CVD risk. The data also provide support and rationale for determining what CVD risk factors to include- based on resource needs. For example, researchers or public health programs may choose to assess WC instead of lipid profile for cardiovascular related problems if ease of assessment and cost are considerations.

## Background

Cardiovascular disease (CVD) is the leading cause of death worldwide, resulting in about 17.9 million deaths, which represents roughly 33% of global deaths [1, 2]. Over the past few decades, the prevalence of obesity and other CVD risk factors in children has been growing in both developing and developed countries [3]. Major reasons for these increases include decreasing levels of in physical activity and high calorie, low nutrient density diets [4]. As a result, CVD, which is currently continuing to increase globally, has become a major public health problem in the 21^st^ century [5].

Unhealthy lifestyle habits are on the rise among children and adolescents, which is contributing to the overall increase in cardiovascular risk among this population [6]. Several prospective analyses have indicated that CVD risk factors in children will tend to carry over into adulthood [5, 7, 8]. While non-modifiable CVD risk factors (e.g., age, sex, and heredity) are unavoidable threats for CVD, whereas modifiable factors such as poor diet, inactivity, smoking, obesity, dyslipidemia, and hypertension, may be changed by implementing lifestyle changes [9]. In response to these concerns, the American Academy of Pediatrics recommends assessing established CVD risk factors, including triglycerides (TG), total cholesterol (TC), body weight, waist circumference (WC), and blood pressure (BP), at selected ages for at-risk individuals in both clinical and public health settings [10, 11].

Even though protocols have been established to assess children and determine whether they are at an elevated risk of CVD, estimating a child’s risk for future CVD morbidity and mortality is difficult. The Framingham Heart Study, which began in 1949, has played an instrumental role in identifying various risk factors (e.g., age, sex, high blood pressure, elevated cholesterol, cigarette smoking, diabetes) and has contributed to creating a risk score for adults that can predict adults risk of developing CVD for the next 10 years [12]. For children, there is no single standardized CVD risk assessment tool. Therefore, researchers from around the world are evaluating the role of multiple individual risk factor variables and interactions between and among multiple risk factors [13–15]; and [16]. Thus, a better understanding of the role and relationships between these CVD risk variables will facilitate assessing CVD risk in children in terms of creating a composite score among these variables or perhaps utilizing a subset of variables that can provide a meaningful tool for the comprehensive evaluation of CVD risk in children in the future.

Although previous studies have calculated composite scores for CVD risk factors based on four or more CVD risk variables, the researchers in those cases did not necessarily justify how they selected the variables they used. For example, Ruiz et al. [16] included 4-6 variables, while Moreira et al. [17] included 6-7 variables in their CVD risk composite scores. These two studies drew upon different variables in creating composite scores; moreover, within each model, the authors likely over weighted higher order factors (e.g., blood lipids per Moreira and colleagues) [18]. In a similar vein, a recent study conducted in 2018 by Stavnsbo et al. [19] explored the relationships among CVD risk variables [body mass index (BMI), WC, systolic blood pressure, diastolic blood pressure, low-density lipoprotein cholesterol (LDL-C), high-density lipoprotein cholesterol (HDL-C),TC to HDL-C ratio, TG, glucose, insulin, homeostatic model assessment score and cardiorespiratory fitness]. In this case, the researchers’ findings suggested an association between cardiorespiratory fitness and improved cardiovascular health in children.

As there is currently no universal standard protocol to assess CVD risk factors in children, deciding which cardiovascular risk variables to include in CVD risk assessment for pediatric populations in public health settings (e.g., schools) remains a topic of debate. Therefore, a better understanding of the interrelationships among variables could guide the selection of a subset of measures that are time, resource, and cost-effective. Ideally, such a subset of measures would adequately estimate a child’s cardiovascular risk. For example, if the relationship between WC and BMI is high, both measures would not need to be assessed, which would substantially reduce time and cost, enabling more efficient screening. Accordingly, the purpose of this study was to explore the relationships among 13 CVD risk factor variables and determine their level of independence. These insights would inform which CVD risk factors to use for cardiovascular screening among children. These variables include resting systolic and diastolic BP, HDL-C, LDL-C, TC, TG, height, weight, WC, BMI, % body fat, acanthosis nigricans, and cardiorespiratory fitness.

## Method

This study employed a cross-sectional study design to evaluate data collected as a part of the (S)Partners for Heart Health project at a Midwestern university in the United States. IRB approval was obtained from the university (No. 07-820), and researchers for the current investigation were all authorized to use the data [20]. Written informed consent was obtained from parents/guardians, and participants were required to assent.

## Participants

Fifth-grade children (girls *n*=856 and boys *n*=669), aged 9–12 years, who took part in the baseline assessment of the (S)Partners for Heart Health from 2010 to 2018 took part in this study. All participants were enrolled in public schools with ≥30% eligibility for free/reduced-price lunch. Standardized pediatric measurement procedures were implemented for this analysis and are summarized below. For a more detailed description of these measures. see Carlson et al. [20].

## Procedure

Measurements for the following variables were obtained by trained undergraduate, graduate, and medical students who demonstrated that they could perform reliable. valid measurements. Specifically, these students were enrolled in an elective course to learn how to perform reliable and valid assessments of CVD risk factors in children and research settings. Each student who performed the measurements had to demonstrate competency by performing the measurements while observed and concurrently measured by study personnel – thus ensuring that the future measurements would be uniform. Given the rigor involved in individually testing all raters, the interrater reliability was not taken into analytical consideration in this study. However, general measurement reliability should be considered in the overall interpretation and application of the results. The variables assessed included resting BP, anthropometric measures, blood lipids, acanthosis nigricans, and cardiorespiratory fitness.

### Anthropometry and body composition

Participants’ height (to the nearest 0.1 cm) and weight (to the nearest 0.1 kg) were assessed twice, and the averaged values were used to calculate BMI ( height in meters squared / body weight in kilograms). Height was measured using a stadiometer (Shorr Production, Olney, MD). Body weight and % body fat were measured using a foot-to-foot bioelectric impedance analysis (BIA) scale (Tanita Corporation, Tokyo, Japan). Waist circumference was measured midway between the lower rib margin and the iliac crest at the end of a gentle expiration [20].

### Blood pressure

Each participant’s resting blood pressure was measured using the manually inflated child-cuff method. Prior to measurement the participants were asked to relax while sitting in an upright position [20]. After sitting for five minutes, systolic and diastolic blood pressure readings were measured twice over a 60-second period. If the readings differed by more than 4 mmHg, a third measure was taken. The mean of the two measures was calculated for both systolic and diastolic blood pressure [20].

### Plasma lipids

Blood samples were taken from each participant’s third or fourth digit. These samples were taken by using a finger prick, collecting the blood into a capillary tube, and then transferring the sample via a plunger onto a cartridge. The blood samples were analyzed using the CardioChek Plus (Polymer Technology Systems Inc. Indianapolis, Indiana, U.S.A) and Cholestech LDX (Cholestech Corporation, Hayward, CA, USA), both of which can a blood sample within 5 minutes. The assay cassettes assessed the levels of TC-C, HDL-C, LDL-C, and TG [20].

### Acanthosis nigricans

Acanthosis nigricans, a condition that is highly related to insulin resistance and type 2 diabetes, is assessed by visual viewing for signs of skin pigmentation and texture changes on various locations on the body including the back of the neck which was the location that the current study used . The children were asked to look at their toes while flexing the neck passively. Measurements (in cm) of pigmentation and texture were assessed in the mid-line identified by the occipital condyle in the cranio-caudal axis (i.e., vertically) from the upper to lower border of pigment, avoiding parallax. A clear ruler was used to measure its length, which was then ranked (negative 0 to severe 4+) [20]. For this study, this measurement was dichotomized as present or non-present to classify the children with acanthosis nigricans.

### Cardiorespiratory fitness

The Progressive Aerobic Cardiovascular Endurance Run (PACER test) was used to evaluate the participants’ cardiorespiratory fitness. An investigator who was (S)Partners protocol-trained explained to the children how the PACER test would be conducted. Participants were instructed to run from one point to a second point at a set distance of 20 m apart while keeping pace with a prerecorded rhythm. The beat was set to music, and the speed was raised each minute by 0.5 km^-^hr^-1^. The participants were instructed to keep up with the rhythm for as long as possible. The test was stopped when a participant was twice unsuccessful in reaching the appropriate marker in the allotted time or was exhausted. The number of laps completed was recorded. The participant’s cardiorespiratory fitness was expressed as ml·kg^−1^·min^−1^ and classified by age according to Fitnessgram recommendations.

### Statistical analysis

Initially, was examined the associations among the various CVD risk factors via bivariate Pearson correlation coefficients. Next, we performed principal component analysis to determine the interdependencies among the variables. Prior to component extraction, parallel analysis was performed to determine the number of components to extract [21] and a varimax orthogonal rotation method was applied to the extracted components. Moreover, principal component analysis was conducted across all participants and stratified by sex to explore any sex differences in component loadings. All analyses were conducted in R; RStudio version 1.1.453 (version 1.1.453 – © 2009-2018 RStudio, Inc. Boston, MA) using the psych package [22].

## Results

Table 1 presents descriptive statistics of each for the CVD risk factor variables. Bivariate Pearson’s correlation coefficients and the variables’ significance levels are shown in Table 2. Most of these variables in Table 2 demonstrated statistically significant correlations with each other, with r values ranging from -0.34 to 0.93.

**Table 1:** Characteristics of the sample population Mean ± SD by gender and combine sample

**Variable**	**n**	**Boys**	**Min-Max**	**n**	**Girls**	**Min-Max**	**n**	**Total Sample**	**Min-Max**
Age (years)	669	10.73±0.50	8.92-14.47	856	10.67±0.48	9.00-13.65	1525	10.7±0.5	8.92-14.47
Total cholesterol (mg/dL)	389	150.01±27.82	100.00-272.00	494	151.03±25.48	100.00-273.00	883	150.6±26.5	100.00-273.00
HDL-cholesterol (mg/dL)	389	50.91±14.37	15.00-99.00	495	48.69±13.22	15.00-101.00	884	49.7±13.8	15.00-101.00
LDL-cholesterol (mg/dL)	298	82.04±25.24	26.00-189.00	433	80.70±24.20	8.00-184.00	731	81.8±24.6	8.00-189.00
Triglycerides (mg/dL)	384	93.73±51.92	45.00-309.00	490	107.86±54.85	45.00-388.00	874	101.65±54.01	45.00-388.00
Height (cm)	629	144.18±6.72	121.43-164.50	832	145.39±7.63	122.30-172.90	1461	144.9±7.8	121.43-172.90
Weight (kg)	624	41.19±11.16	17.20- 87.30	816	43.09±12.56	21.90-97.80	1440	42.3±12.0	17.20-97.80
Body Mass index (kg·m^-2^)	608	19.45±3.88	10.85-34.06	789	19.93±4.28	13.16-33.58	1397	19.7±4.1	10.85-34.06
Body Fat (%)	627	21.61±9.01	5.00-57.00	816	26.03±9.09	5.60-75.05	1443	24.1±9.3	5.00-75.05
Waist circumference (cm)	620	67.30±11.87	36.50-116.75	807	69.41±13.10	25.50-120.30	1427	68.5±12.6	25.50-120.30
Systolic blood pressure (mmHg)	628	103.09±11.04	72.00-139.00	835	104.37±10.66	77.00-135.00	1463	103.8±10.8	72.00-139.00
Diastolic blood pressure (mmHg)	627	66.67±8.76	38.00-90.67	835	67.95±8.85	40.00-100.00	1462	67.4±8.8	38.00-100.00
Acanthosis Nigricans % Present	445	10%		583	14%		1028	12%	
Cardiorespiratory Fitness (mL·kg^-1^·mi^-1^)	423	41.82±6.29	34.62-66.39	536	39.82±3.97	33.58-60.09	959	40.70±5.22	33.58-66.39

**Table 2:** Correlation coefficients of study variables

	1	2	3	4	5	6	7	8	9	10	11	12	13	14	15
1.Gender (1girls – 2 boys)	--														
2. Age (year)	0.06^*^ ^(1525)^	--													
3. Total cholesterol (mmgL)	-0.02 ^(883)^	0.02 ^(871)^	--												
4. HDL- cholesterol (mmgL)	0.08^*^ ^(884)^	0.06 ^(872)^	0.24^**^ ^(877)^	--											
5.LDL-cholesterol (mmgL)	0.01 ^(731)^	-0.02 ^(720)^	0.81^**^ ^(730)^	-0.18^**^ ^(729)^	--										
6. Triglycerides (mgdL)	-0.13^**^ ^(874)^	-0.02 ^(862)^	0.24^**^ ^(867)^	-0.28^**^ ^(869)^	0.00 ^(719)^	--									
7. Height (cm)	-0.08^**^ ^(1461)^	0.28^**^ ^(1428)^	-0.04 ^(865)^	-0.10^**^ ^(866)^	-0.01 ^(715)^	0.09^**^ ^(869)^	--								
8. Weight (kg)	-0.08^**^ ^(1440)^	0.09^**^ ^(1409)^	0.08^*^ ^(859)^	-0.31^**^ ^(860)^	0.18^**^ ^(709)^	0.27^**^ ^(863)^	0.63^**^ ^(1438)^	--							
9.Body mass index (kg·m^-2^)	-0.06^*^ ^(1397)^	-0.02 ^(1389)^	0.10^**^ ^(840)^	-0.34^**^ ^(842)^	0.24^**^ ^(693)^	0.28^**^ ^(844)^	0.33^**^ ^(1397)^	0.93^**^ ^(1397)^	--						
10. Body Fat (%)	-0.23^**^ ^(1443)^	-0.08^*^ ^(1410)^	0.14^**^ ^(867)^	-0.30^**^ ^(867)^	0.24^**^ ^(718)^	0.30^**^ ^(859)^	0.29^**^ ^(1431)^	0.83^**^ ^(1428)^	0.88^**^ ^(1388)^	--					
11.Waist circumference (cm)	-0.08^**^ ^(1427)^	0.04 ^(1396)^	0.13^**^ ^(853)^	-0.33^**^ ^(854)^	0.23^**^ ^(703)^	0.32^**^ ^(857)^	0.45^**^ ^(1425)^	0.91^**^ ^(1414)^	0.92^**^ ^(1372)^	0.87^**^ ^(1406)^	--				
12.Systolic blood pressure (mmHg)	-0.06^*^ ^(1463)^	0.07^**^ ^(1428)^	0.06 ^(875)^	-0.07^*^ ^(876)^	0.07^*^ ^(723)^	0.07^*^ ^(866)^	0.25^**^ ^(1439)^	0.34^**^ ^(1420)^	0.31^**^ ^(1377)^	0.31^**^ ^(1424)^	0.29^**^ ^(1408)^	--			
13.Diastolic blood pressure (mmHg)	-0.07^**^ ^(1462)^	0.01 ^(1427)^	0.08^*^ ^(875)^	-0.12^**^ ^(876)^	0.13^**^ ^(723)^	0.06 ^(866)^	0.17^**^ ^(1438)^	0.25^**^ ^(1419)^	0.23^**^ ^(1376)^	0.26^**^ ^(1423)^	0.24^**^ ^(1407)^	0.57^**^ ^(1462)^	--		
14.Acanthosis Nigricans	-0.05 ^(1028)^	-0.01 ^(1002)^	0.05 ^(589)^	-0.17^**^ ^(590)^	0.12^**^ ^(492)^	0.12^**^ ^(579)^	0.24^**^ ^(1012)^	0.48^**^ ^(998)^	0.42^**^ ^(965)^	0.44^**^ ^(1005)^	0.47^**^ ^(986)^	0.11^**^ ^(1014)^	0.14^**^ ^(1014)^	--	
15.Cardiorespiratory Fitness (mL^.^kg^-1.^min^-1)^	0.19^**^ ^(959)^	0.000 ^(956)^	-0.09^*^ ^(616)^	0.11^**^ ^(611)^	-0.10^*^ ^(500)^	-0.13^**^ ^(613)^	-0.12^**^ ^(949)^	-0.32^**^ ^(938)^	-0.34^**^ ^(928)^	-0.38^**^ ^(933)^	-0.30^**^ ^(936)^	-0.16^**^ ^(938)^	-0.09^**^ ^(938)^	-0.14^**^ ^(620)^	--

^*^p < .05, **p < .01, two-tailed. The sample size in parentheses under coefficient

Parallel analysis indicated that two components should be extracted, which explained about 52% of the total variance among 10 CVD risk variables included in the analysis. Out of 13 total CVD risk variables, only 8 were included in the component analysis. Five variables (age, weight, height, gender, and TC) were excluded from the principal component analysis, as these variables were not previously used as independent CVD risk indicators or not independent (i.e., TC is a combination of other cholesterols). Among the two components extracted, principal component 1 accounted for 34% of the total variance and variables including WC, % body fat, BMI, acanthosis nigricans, cardiorespiratory fitness, and TG loaded highest (>.40). Principal component 2 accounted for an additional 17% of the total variance; the variables that including loaded highest (>.80) were systolic blood pressure and diastolic blood pressure. Factor loadings were consistent across gender. See Table 3 for details.

**Table 3:** Rotated (varimax) principal component loadings of CVD risk factors across all participants and stratified by sex

**Variable**	Principal component (total sample)		Boys		Girls	
	1	2	1	2	1	2
Body Mass index	**0.87**	0.28	**0.81**	0.33	**0.90**	0.29
Waist circumference	**0.89**	0.24	**0.87**	0.30	**0.90**	0.25
Body Fat	**0.88**	0.27	**0.84**	0.34	**0.89**	0.27
Acanthosis Nigricans	**0.57**	0.08	**0.48**	0.39	**0.61**	-0.02
Diastolic blood pressure	0.08	**0.85**	0.00	**0.85**	0.07	**0.86**
Systolic blood pressure	0.13	**0.86**	0.13	**0.80**	0.09	**0.88**
HDL-cholesterol	**-0.51**	0.11	**-0.43**	0.09	**-0.56**	0.08
Triglycerides	**0.45**	-0.15	**0.52**	-0.20	**0.41**	-0.08
LDL-cholesterol	0.31	0.13	0.30	0.30	0.28	0.05
Cardiorespiratory Fitness	**-0.43**	-0.14	**-0.45**	-0.13	**-0.41**	-0.15
SS Loading	3.42	1.74	3.11	1.93	3.52	1.76
Proportion variance	34%	17%	31%	19%	35%	18%
Cumulative variance	34%	52%	31%	50%	35%	53%

Loadings ≥0.40 are in bold type

## Discussion

The overall purpose of this study was to explore the interrelations among various CVD risk indicators in adolescents. Among the diverse set of CVD risk factors assessed in the current study, the strength of the relationships varied widely. The findings suggest that two main components can explain roughly 50% of the variability in the data, with adiposity and blood pressure being the dominant indicators. Our results align with those of several adult studies that have used principal component analysis across various cardiovascular risk variables [18, 23, 24]. For example, while those studies included some measures of insulin and glucose, they also revealed separate components related to adiposity, dyslipidemia (but specifically HDL and TG), and BP. Specifically, out of the four components extracted in our data, one comprised adiposity and cardiorespiratory fitness, one involved blood pressure measures, and one highlighted HDL and TG; meanwhile, in the last component, LDL loaded highest. These results are nearly identical to Cox et. al.’s findings [19] The difference in the number of components extracted between studies is likely based on the different methods used; in particular, [19] involved extracting components with eigenvalues greater than or equal to 1,in contrast to the parallel analysis method that we used [25]. Despite these minor analytical differences, the similarities between our child focused and the previous adult component analysis, as well as previous prospective studies [5, 7, 8], suggest that the assessment of CVD risk in children may be similar to that for adults.

The findings of the current study have several implications. First, the creation of a simple summative CVD risk composite should consider the variables used to avoid overweighting any particular component. For instance, some studies [25–27] have included both BMI and WC in composite scores thus overweighting the “adiposity” component. Second, from a practical perspective, the data also provide information that justifies selected CVD indicators. For example, the apparently strong relationship among various measures of adiposity may support future researchers choice of the easiest/least intrusive way to measure this factor (e.g., BMI). Additionally, health practitioners wishing to obtain a good overall representation of CVD risk with a minimal number of variables may simply need to choose one adiposity measure (e.g., BMI), given the relationship and high factor loading. Lastly, adiposity measures seem to load highly on a component that explains a large amount of variability in the data, highlighting the importance of assessing and mitigating obesity.

Another CVD risk variable that is commonly associated with childhood obesity and hyperinsulinemia in children is acanthosis nigricans. In this study, acanthosis nigricans exhibited a factor loading of 0.57 on the first extracted principal component, and revealed a strong positive correlation with other adiposity measures such as weight (0.48), BMI (0.42), body fat (0.44), and WC(0.47), (as can be seen in Table 2). As acanthosis nigricans is an early indicator of insulin resistance and underlying diseases in children and adolescence, it may be worthwhile to include this factor among the CVD risk assessment variables to be assessed in this population. Moreover, some previous studies have highlighted a higher prevalence of acanthosis nigricans among minority populations (Hispanic, African American etc.), and obese children [28–31]. Lastly, acanthosis nigricans should not be underestimated for its utility in CVD screening, since it acts as a cutaneous marker of underlying diseases and is relatively quick and simple to measure [32].

Meanwhile, a blood lipid profile includes numerous variables that are considered important markers for the identification of CVD risk. The National Heart, Lung, and Blood Institute (NHLBI) recommends a comprehensive evaluation of serum lipids and lipoproteins for children and adolescents with an abnormal lipid profile [33]. In our study, HDL-C showed a high factor loading of -0.51 on the first extracted principal component, indicating an interrelationship with other CVD risk factors that explain a substantial amount of the variability in the data. Several studies have reported that a low level of HDL-C is a risk factor for the development of CVD [34–37]. Our data demonstrated that adiposity variables had a moderate negative correlation with HDL-C (-0.31 to - 0.34), (as shown in Table 2). In a similar vein, the NHLBI documented an association between both dyslipidemia patterns and the initiation and progression of atherosclerotic lesions in children and adolescents as demonstrated by pathology and imaging studies [33]. Lastly, HDL-C has anti-inflammatory, antioxidant, and anti-thrombotic properties, all of which are excellent assets for minimizing CVD risks [36].

Another CVD risk variable that had a higher factor loading on the first extracted component was TG, with a factor loading of 0.45. Based on the NHLBI cut-off values, our data also demonstrated a higher prevalence of high TG levels among the children investigated in this study (Table 1). Other cardiovascular-related studies have also highlighted the independent role of TG in the development of CVD [38–40]. For example, according to Berenson and colleagues, the clustering of lipid risk factors, such as an increase in TG and other risk factors, was significantly linked to the development of atherosclerotic lesions related to fatty streaks and fibrous plaques at a young age [34]. Similarly, Morrison and colleagues confirmed that childhood TG levels were highly related to the development of young adult CVD [41]. Based on the findings of these previous studies and higher loadings on the principal component within the current study, TG may be a useful indicator of CVD risk assessment in children and adolescents.

Cardiorespiratory fitness was another CVD risk variable that loaded highly on the first component. This factor revealed a moderate correlation with other adiposity variables including weight, BMI, and WC(Table 2). Previous studies in children have also suggested that a low cardiorespiratory fitness level is associated with a high clustering of CVD risk factors [15, 42–44], which can ultimately lead to CVD. In contrast, a recent longitudinal study indicated that a high level of cardiorespiratory fitness might protect children from being obese and developing hypertension later in life [45]. Moreover, cardiorespiratory fitness involves a non-invasive measure that can be assessed in field settings with minimal equipment and provides very useful information for CVD assessment.

Systolic and diastolic blood pressure loaded highly on the second extracted component with factor loadings of 0.86 and 0.85, respectively.. Previous studies have included both systolic and diastolic blood pressure into their composite scores which might have led to overweighting of a higher order factor [16, 17]. Children who have higher blood pressure can develop prehypertension which can lead to hypertension when they become adults [46]. However, in the current study, blood pressure was relatively independent of other variables (see Table 2). Furthermore, an increase in childhood systolic blood pressure has been linked to changes in arterial thickness that can lead to left ventricular enlargement in adults [47]. For these reasons, assessment of blood pressure (systolic or diastolic) at an early age is recommended for inclusion in screening for CVD risk in children.

The current study has two main strengths. First, the study included a diverse set of CVD risk variables in its analysis. Second, as no standard definitions are currently available for the calculation of CVD risk in children, the study findings provide information regarding the selection of variables, especially for pediatric field studies in non-clinical locations such as schools and out of hospital settings. However, the present study was limited by its cross-sectional design, which prevented the assessment of these CVD risk factors over time. Thus, future longitudinal studies are warranted to examine the pathological and physiological effects of varying levels of CVD risk indicators in children.

## Conclusion

The current study identified relationships among a variety of CVD risk factors in children. According to the study findings, adiposity and blood pressure showed the most independence and explained the most variability, meaning that it may not be necessary to assess large numbers of cardiovascular risk factors in field-based public health settings where resources are a concern. Although some of the blood lipid measures and cardiorespiratory fitness loaded high on the adiposity factor, the strength of association was not as high as that for some of the other variables, indicating more independence. Thus, future studies seeking to thoroughly assess relatively independent CVD indicators may benefit from utilizing blood lipids and cardiorespiratory fitness measures. In conclusion, researchers should consider using these findings in future studies for more efficient and effective CVD risk assessment and to increase CVD prevention efforts among children.

## Data Availability

The datasets used and analyzed during the current study are available from the corresponding author on reasonable request.

## Abbreviations

CVD: Cardiovascular disease
LDL: Low-density lipoprotein
HDL: High-density lipoprotein
TC: Total cholesterol
BP: Blood pressure
BMI: Body mass index
PACER: The Progressive Aerobic Cardiovascular Endurance Run
